# Treatment outcome of tuberculosis patients at Gondar University Teaching Hospital, Northwest Ethiopia. A five - year retrospective study

**DOI:** 10.1186/1471-2458-9-371

**Published:** 2009-10-04

**Authors:** Belay Tessema, Abebe Muche, Assegedech Bekele, Dieter Reissig, Frank Emmrich, Ulrich Sack

**Affiliations:** 1 Department of Medical Laboratory Technology, College of Medicine and Health Sciences, University of Gondar, Gondar, Ethiopia; 2 Department of Anatomy, College of Medicine and Health Sciences, University of Gondar, Gondar, Ethiopia; 3 Institute of Anatomy, Faculty of Medicine, University of Leipzig, Leipzig, Germany; 4 Institute of Clinical Immunology and Transfusion Medicine, Faculty of Medicine, University of Leipzig, Leipzig, Germany; 5 Institute of Medical Microbiology and Epidemiology of Infectious Diseases, Faculty of Medicine, University of Leipzig, Leipzig, Germany; 6 Fraunhofer Institute for Cell Therapy and Immunology, Leipzig, Germany

## Abstract

**Background:**

In Gondar University Teaching Hospital standardized tuberculosis prevention and control programme, incorporating Directly Observed Treatment, Short Course (DOTS) started in 2000. According to the proposal of World Health Organization (WHO), treatment outcome is an important indicator of tuberculosis control programs. This study investigated the outcome of tuberculosis treatment at Gondar University Teaching Hospital in Northwest Ethiopia.

**Methods:**

We analyzed the records of 4000 tuberculosis patients registered at Gondar University Teaching Hospital from September 2003 to May 2008. Treatment outcome and tuberculosis type were categorized according to the national tuberculosis control program guideline. Multivariate analysis using logistic regression model was used to analyse the association between treatment outcome and potential predictor variables.

**Results:**

From the total of 4000 patients, tuberculosis type was categorized as extrapulmonary in 1133 (28.3%), smear negative pulmonary tuberculosis in 2196 (54.9%) and smear positive pulmonary tuberculosis in 671 (16.8%) cases. Of all patients, treatment outcome was classified as successfully treated in 1181(29.5%), defaulted in 730 (18.3%), died in 403 (10.1%), treatment failed in six (0.2%) and transferred out in 1680 (42.0%) patients. Males had the trend to be more likely to experience death or default than females, and the elderly were more likely to die than younger. The proportion of default rate was increased across the years from 97(9.2%) to 228(42.9%). Being female, age group 15-24 years, smear positive pulmonary tuberculosis and being urban resident were associated with higher treatment success rate.

**Conclusion:**

The treatment success rate of tuberculosis patients was unsatisfactorily low (29.5%). A high proportion of patients died (10.1%) or defaulted (18.3%), which is a serious public health concern that needs to be addressed urgently.

## Background

Tuberculosis (TB) is the leading cause of death from a curable infectious disease [1]. According to the 2007 report of World Health Organization (WHO), one-third of the world's population is estimated to be infected with tubercle bacilli and hence at risk of developing active disease, in 2005, the annual incidence of TB, expressed as the number of new TB cases, was globally about 8.8 million people (7.4 million of these were in Asia and sub-Saharan Africa), and the annual number of deaths due to TB was 1.6 million, including 195,000 patients infected with HIV [2].

Important challenges for TB control are human immunodeficiency virus (HIV) co-infection and drug resistance [3,4]. HIV co-infection is the strongest known risk factor for progression of latent TB infection to TB disease [5]. Although HIV co-infection has been shown not to affect the failure rate of TB treatment, high mortality has been reported among HIV-infected TB patients in sub-Saharan Africa [6].

According to the 2008 report of WHO, Ethiopia ranks seventh among the world's 22 countries with a high tuberculosis burden [7]. The Ethiopian Federal Ministry of Health (FMOH) hospital statistics data has shown that tuberculosis is the leading cause of morbidity, the third cause of hospital admission (after deliveries and malaria), and the second cause of death in Ethiopia, after malaria [8]. Based on the 2007 WHO estimates, in Ethiopia, the incidence of TB of all forms and smear positive TB stand at 341 and 152 per 100,000 population, respectively. The prevalence and mortality of tuberculosis of all forms is estimated to be 546 and 73 per 100,000 population respectively [2].

In Ethiopia a standardized TB prevention and control programme, incorporating Directly Observed Treatment, Short Course (DOTS), was started in 1992 as a pilot in Arsi and Bale zone, Oromia Region. The DOTS strategy has been subsequently scaled up in the country and implemented at national level. DOTS as a strategy was introduced to the tuberculosis control programme at Gondar University Teaching Hospital in 2000. Currently the DOTS geographic coverage reaches 90%, whereas the health facility coverage is 75% [8]. Like other developing countries, in Ethiopia, culture and drug susceptibility testing for *M. tuberculosis *are not performed routinely; even for patients suspected of harboring drug-resistant strains.

DOTS during at least the first 2 months of treatment, in which patients take drugs directly under the observation of health care providers, has been recommended by international tuberculosis authorities[9,10], and has been shown to be effective in achieving a high successful treatment rate, from 86% to 96.5% [11]. The utility of DOTS has also been demonstrated in developed countries. Jasmer *et al *reported that DOTS was significantly associated with a higher treatment success rate than self-administered therapy (97.8% *vs*. 88.6%, *p *< 0.002) and a lower tuberculosis-related mortality rate (0% *vs*. 5.5%, *p *= 0.002) [12]. DOTS also leads to significant reductions in the frequency of primary drug resistance, acquired drug resistance and relapse [13].

Correct treatment of tuberculosis aims at curing the patient, interrupting transmission of tuberculosis to other persons and preventing bacilli from becoming drug resistant. These aims are not achieved in many regions of the world even when anti tuberculosis drugs are available [14]. The main reasons are death of the patients during treatment, default before the scheduled end of treatment or resistance to the drugs prescribed. Patient non-adherence to treatment is interpreted as a failure of the health care system to cope with the natural tendency of humans to quit treatment as soon as they feel subjectively better, or better without treatment if adverse drug events supervene [12].

Treatment outcome results serve as a proxy of the quality of TB treatment provided by a health care system. Recommendations on how to evaluate treatment outcomes using standardized categories have been issued by the World Health Organization (WHO) in conjunction with the European Region of the International Union Against Tuberculosis and Lung Disease (IUATLD) [15]. These categories were defined to assess the risk of future relapse and drug resistance. Ideally, treatment outcome in all patients should be routinely monitored by the epidemiological surveillance system. This would make it possible to recognize and amend system failures before the incidence and proportion of resistant isolates rise. However, treatment outcome of tuberculosis patients has not been assessed yet in northwest Ethiopia. Therefore, this study is aimed to assess treatment outcomes of pulmonary tuberculosis and extrapulmonary tuberculosis cases at Gondar University Teaching Hospital, Northwest Ethiopia.

## Methods

### Study setting

Gondar University Teaching Hospital (GUTH) is a tertiary health care hospital serving the population of Gondar town and remote hilly areas of northwest Ethiopia. The total population served by the hospital is about 5 million. In the hospital a Directly Observed Therapy; Short-Course (DOTS) clinic is operating under the National Tuberculosis and Leprosy Control Program (NTLCP) of Ethiopia, under which the diagnosis of pulmonary TB is followed by examination of three sputum smears by Zihel -Nielsen staining method for acid fast bacilli (AFB). Chest radiographs and pathological investigations are also used to support the diagnosis. Patients diagnosed with tuberculosis are referred to the DOTS clinic where they are registered and treated according to the national TLCP guideline [8].

### Study design and data collection

A retrospective analysis of the profile and treatment outcome of all tuberculosis patients registered from September 1, 2003 to May 31, 2008 at DOTS Clinic was conducted. The registration documents reviewed contain basic information such as patient's age, sex, address, tuberculosis type, and treatment outcome. Institutional ethical clearance was obtained from the Research and Publication Committee of Gondar University.

### Definition

According to the standard definitions of the National Tuberculosis and Leprosy Control Program guideline (NLCP) adopted from WHO [8], the following clinical case and treatment outcome definitions were used:

### Pulmonary TB, smear-positive

A patient with at least two sputum specimens which were positive for acid-fast bacilli (AFB) by microscopy, or a patient with only one sputum specimen which was positive for AFB by microscopy, and chest radiographic abnormalities consistent with active pulmonary TB.

### Pulmonary TB, smear-negative

A patient with symptoms suggestive of TB, with at least two sputum specimens which were negative for AFB by microscopy, and with chest radiographic abnormalities consistent with active pulmonary TB (including interstitial or miliary abnormal images), or a patient with two sets of at least two sputum specimens taken at least two weeks apart, and which were negative for AFB by microscopy, and radiographic abnormalities consistent with pulmonary TB and lack of clinical response to one week of broad spectrum antibiotic therapy.

### Extrapulmonary TB (EPTB)

This included tuberculosis of organs other than the lungs, such as lymph nodes, abdomen, genitourinary tract, skin, joints and bones, meninges, etc. Diagnosis of EPTB was based on fine needle aspiration cytology or biochemical analyses of cerebrospinal/pleural/ascitic fluid or histopathological examination or strong clinical evidence consistent with active extrapulmonary tuberculosis, followed by a decision of a clinician to treat with a full course of anti-tuberculosis chemotherapy. In all the cases of EPTB, sputum examinations and chest radiographs were used to investigate the involvement of lung parenchyma. This hospital lacks the facilities for culture and drug susceptibility testing.

### Treatment Outcome

The treatment outcome was divided into seven categories according to NTLCP guideline. These categories were: *cured *(finished treatment with negative bacteriology result at the end of treatment), *completed treatment *(finished treatment, but without bacteriology result at the end of treatment), *failure *(remaining smear positive at five months despite correct intake of medication), *defaulted treatment *(patients who interrupted their treatment for two consecutive months or more after registration), *died *(patients who died from any cause during the course of treatment), *transferred out *(patients whose treatment results are unknown due to transfer to another health facility) and successfully treated(A patient who was cured or completed treatment).

### Statistical analysis

Data were entered, cleared and analysed using the statistical package SPSS for windows, version 13. To ensure the quality of data entered into the computer, two people were independently cross-checked each entry. For categorical data, we used proportions with 95% confidence intervals, Odds ratio and Chi-square test to compare different groups. Multivariate analysis using logistic regression model was used to analyse the association between treatment outcome and potential predictor variables. P values of less than 0.05 were considered statistically significant.

## Results

### Demographic characteristics of patients

A total of 4000 tuberculosis patients were registered at Gondar University Teaching Hospital between September 2003 and May 2008. Of these, 2140(53.5%) were males and 1860 (46.5%) were females with the mean (SD) age of 27.7(15.2) years. Fifty one point five percent (n = 2062) of the patients were urban resident and 2196 (54.9%) patients were smear negative pulmonary tuberculosis. Table 1 shows the general characteristics of the patients. The proportion of tuberculosis types across the years are shown in Table 2, where the number of smear negative pulmonary tuberculosis cases (53.9%-56.5%) remained highest compared to smear positive pulmonary tuberculosis cases (15.8%-17.9%) and extrapulmonary tuberculosis cases(25.6% - 30.1%) over the years.

**Table 1:** General characteristics of study subjects (n = 4000), Gondar University Teaching Hospital, 2003-2008.

**Characteristics**	**Frequency**	**Percent**
**Sex**		
Male	2140	53.5
Female	1860	46.5
**Address**		
Urban	2062	51.5
Rural	1938	48.5
**Age group(years)**		
0-14	747	18.7
15-24	901	22.5
25-34	1142	28.6
35-44	672	16.8
45-54	297	7.4
55-64	142	3.5
≥ 65	99	2.5
**Tuberculosis type**		
Smear positive pulmonary TB	671	16.8
Smear negative pulmonary TB	2196	54.9
Extrapulmonary TB	1133	28.3

TB = tuberculosis

**Table 2:** Trends of treatment outcome and tuberculosis type across the years for all cases of tuberculosis, Gondar University Teaching Hospital, 2003-2008.

**Treatment outcome & tuberculosis type**	**Years**					**Total** **N (%)**
		
	**Sept.2003 - Aug.2004** **N (%)**	**Sept.2004 - Aug.2005** **N (%)**	**Sept.2005 -Aug.2006** **N (%)**	**Sept.2006 - Aug.2007** **N (%)**	**Sept.2007 - May 2008** **N (%)**	
**Treatment outcomes**						
Treatment success	321(30.5)	200(28.9)	390(35.3)	213(34.3)	57(10.7)	**1181(29.5)**
Transferred out	488(46.4)	317(45.9)	431(39.0)	226(36.4)	218(41.0)	**1680(42.0)**
Default	97(9.2)	81(11.7)	165(14.9)	159(25.6)	228(42.9)	**730(18.3)**
Death	146(13.9)	93(13.5)	116(10.5)	21(3.4)	27(5.1)	**403(10.1)**
Failure	0(0.0)	0(0.0)	2(0.2)	2(0.3)	2(0.4)	**6(0.2)**
**Tuberculosis types**						
Smear positive pulmonary TB	172(16.3)	109 (15.8)	189(17.1)	111(17.9)	90(16.9)	**671(16.8)**
Smear negative pulmonary TB	583(55.5)	374(54.1)	601(54.4)	351 (56.5)	287(53.9)	**2196(54.9)**
Extrapulmonary TB	297(28.2)	208(30.1)	314(28.4)	159(25.6)	155 (29.1)	**1133(28.3)**

TB = tuberculosis, Sept = September, Aug = August, N = number

### Treatment outcome

We analyzed treatment outcome of 4000 tuberculosis patients who were registered at the hospital during the study period. Of these, 1181(29.5%) had successfully treated, 730 (18.3%) defaulted, 403 (10.1%) died, 6(0.2%) treatment failure and 1680 (42.0%) transferred out. The death rate of tuberculosis patients was steadily decreased over the study period from 146(13.9%) in (September 2003 - August 2004) to 93(13.5%) in (September 2004 - August 2005), 116(10.5%) in (September 2005 - August 2006), 21(3.4%) in (September 2006 - August 2007) and 27(5.1%) in (September 2007 - May 2008). However, default rate was increased across the years from 97(9.2%) in (September 2003 - August 2004) to 81(11.7%) in (September 2004 - August 2005), 165(14.9%) in (September 2005 - August 2006), 159(25.6%) in (September 2006 - August 2007) and 228(42.9%) in (September 2007 - May 2008) (table 2).

Table 3 shows that as the age of tuberculosis patients increased, death rate of patients was increased from 34(4.6%), to 64(7.1%), 138(12.1%), 98(14.6%), 47(15.8%), 12(15.8%) and 10(10.1%) in the age groups of 0 - 14 years, 15 - 24 years, 25 - 34 years, 35 - 44 years, 45 - 54 years, 55 - 64 years and > 65 years respectively. In addition, males had the trend to be more likely to experience death (OR = 1.12, 95% CI = 0.91 - 1.38) or default (OR = 1.15, 95% CI = 0.98 - 1.35) than females as an outcome.

**Table 3:** Treatment outcome by sex, address, age group and tuberculosis type, Gondar University Teaching Hospital, 2003-2008.

**Characteristics**	**Treatment outcomes**					**Total**
		
	**Treatment success** **N(%)**	**Transferred out** **N (%)**	**Default** **N (%)**	**Death** **N (%)**	**Failure** **N (%)**	
**Sex**						
Male	577(27.0)	921(43.0)	411(19.2)	226(10.6)	5(0.2)	2140
Female	604(32.5)	759(40.8)	319(17.2)	177(9.5)	1(0.1)	1860
**Address**						
Urban	867(42.0)	590(28.6)	379(18.4)	222(10.8)	4(0.2)	2062
Rural	314(16.2)	1090(56.2)	351(18.1)	181(9.3)	2(0.1)	1938
**Age group(years)**						
0-14	168(22.5)	439(58.8)	106(14.2)	34(4.6)	0(0.0)	747
15-24	373(41.4)	310(34.4)	151(16.8)	64(7.1)	3(0.3)	901
25-34	334(29.2)	433(37.9)	236(20.7)	138(12.1)	1(0.1)	1142
35-44	176(26.2)	260(38.7)	136(20.2)	98(14.6)	2(0.3)	672
45-54	74(24.9)	132(44.4)	44(14.8)	47(15.8)	0(0.0)	297
55-64	34(24.9)	63(44.4)	33(14.8)	12(15.8)	0(0.0)	142
≥ 65	22(22.2)	43(43.4)	24(24.2)	10(10.1)	0(0.0)	99
**Tuberculosis type**						
Smear positive pulmonary TB	321(47.8)	195(29.1)	105(15.6)	46(6.9)	4(0.6)	671
Smear negative pulmonary TB	564(25.7)	987(44.9)	404(18.4)	239(10.9)	2(0.1)	2196
Extrapulmonary TB	296(26.1)	498(44.0)	221(19.5)	118(10.4)	0(0.0)	1133

TB = tuberculosis, N = number

As shown in table 4, female tuberculosis patients had significantly higher treatment success rate (32.5% vs. 27.0%; p = 0.01) than males. Patients from rural areas, had significantly lower treatment success rate compared to cases from urban (16.2% vs. 42%; p < 0.001). Furthermore, patients in the age group of 25 - 34 years had significantly low treatment success rate compared to other age groups (P = 0.002; CI = 0.26 - 0.74).

**Table 4:** Crude and Adjusted odds ratios for various factors that might affect treatment outcome among tuberculosis patients, Gondar University Teaching Hospital, 2003-2008.

**Characteristics**	**Treatment success**		**Crude OR**	**95% CI**	**P-Value**	Adjusted OR*	**95% CI**	**P-Value**
							
	**Yes** **N (%)**	**No** **N (%)**						
**Sex**								
Male	577(27.0)	1563(73.0)	1.00	---	---	1.00	---	---
Female	604(32.5)	1256(67.5)	1.303	1.137-1.493	<0.001	1.215	1.048-1.409	0.010
**Address**								
Urban	867(42.0)	1195(58.0)	1.00	---	---	1.00	---	---
Rural	314(16.2)	1624(83.8)	0.266	0.230-0.309	<0.001	0.275	0.235-0.322	<0.001
**Age group(yrs)**								
0-14	168(22.5)	579(77.5)	1.00	---	---	1.00	---	---
15-24	373(41.4)	528(58.6)	0.985	0.595-1.630	0.952	0.697	0.409-1.188	0.184
25-34	334(29.2)	808(70.8)	0.404	0.247-0.662	<0.001	0.437	0.259-0.736	0.002
35-44	176(26.2)	496(73.8)	0.691	0.423-1.129	0.140	0.770	0.458-1.293	0.323
45-54	74(24.9)	223(75.1)	0.805	0.486-1.333	0.400	0.832	0.489-1.418	0.499
55-64	34(23.9)	108(76.1)	0.861	0.501-1.480	0.588	0.941	0.531-1.667	0.834
≥ 65	22(22.2)	77(77.8)	0.908	0.493-1.672	0.756	0.934	0.490-1.780	0.835
**TB type**								
Smear positive pulmonary TB	321(47.8)	350(52.2)	1.00	---	---	1.00	---	---
Smear negative pulmonary TB	564(25.7)	1632(74.3)	0.386	0.315-0.472	<0.001	0.430	0.345-0.535	<0.001
Extrapulmonary TB	296(26.1)	837(73.9)	1.023	0.869-1.205	0.782	1.019	0.856-1.213	0.831

TB = tuberculosis, OR = Odds Ratio, CI = Confidence interval, N = number, yrs = years

* = All the variables in the table are included in the model.

Smear negative pulmonary tuberculosis patients had significantly low treatment success rate (P < 0.001; CI = 0.35 - 0.54) compared to smear positive and extrapulmonary tuberculosis patients. On the other hand, the highest treatment success rate was observed among smear positive pulmonary tuberculosis patients 321(47.8%) compared to smear negative pulmonary tuberculosis and extrapulmonary tuberculosis patients 564(25.7%) and 296(26.1%) respectively. In addition, the highest treatment success rate (35.3%; p < 0.001; CI = 0.24 - 0.46) was observed from September 2005 to August 2006 compared to treatment success rates across the years during the study period (table 4).

## Discussion

According to the WHO 2005 report on global tuberculosis control [16], the treatment success rates under the DOTS programs among 22 high-burden countries (HBCs) varied from 60% in Uganda to 93% in China, with an average of 83%. Moreover, the study conducted by B Shargie et al., in Southern Ethiopia has shown that the treatment success rate of all tuberculosis cases was 49% [17]. Our study found that the treatment outcome of tuberculosis patients treated under DOTS program at Gondar University Teaching Hospital was unsatisfactory. The treatment success rate of all tuberculosis cases was 29.5%. The low treatment success rate observed in this study might be due to high transferred out rate (42%), default rate (18.3%) and death rate (10.1%), but the high treatment default rate and death rate deserve special attention.

This study also demonstrated that death rate of tuberculosis patients was significantly decreased across the years from13.9% in (September 2003 - August 2004) to 3.4% in (September 2006 - August 2007) (p < 0.001). This might be due to increasing efforts to encourage tuberculosis patients for HIV screening and initiating anti retroviral therapy for TB/HIV co - infected patients at this hospital. Patient registration documents we reviewed lack information about the HIV status of our study subjects. However, a previous study conducted by Kassu et al showed that high proportions (52.1%) of TB patients were co-infected with HIV at Gondar University Hospital [18].

As shown in table 3, we analyzed the death rate across the age groups of tuberculosis patients and as the age increase the death rate of patients was steadily increased from 4.6% in the age group of 0 - 14 years to 15.8% in the age group of 55 - 64 years. This is in agreement with the finding of a study conducted by Lee et al [19]. High age has been previously reported to be a risk factor for death, partly due to increasing comorbidities as well as the general physiological deterioration with age [20-22], because of which close monitoring of treatment in older patients is necessary.

The default rate in this study (18.3%) was higher than the average 6.2% among the 22 HBCs [16] and 10% among the rural households in northwest Ethiopia [23]. On top of that default rate of tuberculosis patients was significantly (p < 0.001) increased across the years from 97(9.2%) in (September 2003 - August 2004) to 228(42.9%) in (September 2007 - May 2008) (Table 2). According to studies conducted in central India [24] and in Malawi [25] patients who completed treatment had a better understanding of the duration of TB treatment than patients who interrupted treatment. In other settings, counseling [26], better supervision [27,28], home visits and motivation [29,30] and health education [31] have been used successfully as interventions to reduce default rate of tuberculosis patients. The complementary results obtained from the quantitative and qualitative components of the study conducted in northwest Ethiopia also indicate that the TB club approach has a significant impact in improving patients' compliance to anti-TB treatment and in building positive attitudes and practice in the community regarding TB [32]. Thus, the finding of this study indicating the necessity of strengthening defaulter tracing and interventions to reduce default rate of tuberculosis patients in the study area.

The treatment failure rate varied from 0.1% in Zimbabwe to 9.1% in the Russian Federation, with an average of 1.5% in HBCs [16]. The treatment failure rate in this study was 0.2%, which is lower than the average failure rate of the HBCs. This might be due to lower prevalence of multi drug resistant strains of *M. tuberculosis *at the study area.

In our study the number of smear negative pulmonary tuberculosis cases (53.9% -56.5%) remained highest compared to smear positive and extrapulmonary tuberculosis cases over the years. Similarly, registration in DOTS (directly observed therapy, short course) programme areas in Addis Ababa, Ethiopia has shown a continuous increase in the proportion of smear-negative pulmonary TB from 36.3% in 1992 to 66.3% in 1999 (G. Fishay, TB co-ordinator, National Tuberculosis and Leprosy Control Programme, personal communication, 1999). The large number of smear negative pulmonary tuberculosis cases might be due to high proportion of TB - HIV co-infection at the study area, as shown by a previous study [18]. HIV-infected patients are twice as likely to have sputum smear-negative, culture-positive pulmonary TB (PTB) [33-35]. This results from their compromised immune response causing less cavity formation [36].

The main limitations of our study were selection bias and lack of information about the HIV status of the study subjects. Nevertheless, our study reveals that TB is still a major public health problem in Gondar University Teaching Hospital. The results of the present study also indicate a need for a coordinated tuberculosis control program which should include active case surveillance, effective care and treatment and quality laboratory diagnosis services. Therefore, physicians should be encouraged to assess HIV risk factors in patients who present with TB and to offer HIV testing to all TB patients, to document the HIV status of TB patients at TB clinic and to monitor treatment responses. Furthermore, efforts should be made to improve the quality of the laboratory diagnosis services.

## Conclusion

The treatment success rate of pulmonary and extrapulmonary tuberculosis patients treated at Gondar University Teaching Hospital in northwest Ethiopia was unsatisfactorily low (29.5%). A high proportion of patients died (10.1%) or defaulted (18.3%), which is a serious public health concern that needs to be addressed urgently. To improve treatment outcome of tuberculosis patients we recommend enhanced supervision and monitoring, improved counseling during the intensive and continuation phases of treatment, home visits and motivation of patients (defaulter tracing) and health education to reduce treatment interruption.

## Competing interests

The authors declare that they have no competing interests.

## Authors' contributions

**BT **was the primary researcher, conceived the study, designed, participated in data collection, conducted data analysis and drafted the manuscript for publication. **AM** and **AB **assisted in data collection and preparation of first draft of manuscript. **DR, FE **and **US **interpreted the results, and reviewed the initial and final drafts of the manuscript. All authors read and approved the final manuscript.

## Pre-publication history

The pre-publication history for this paper can be accessed here:


